# Clinical and cost-effectiveness of individualised exercises and foot orthoses in the treatment of plantar heel pain: protocol for the TREADON randomised multi-arm multi-stage adaptive trial

**DOI:** 10.3310/nihropenres.13930.1

**Published:** 2025-05-06

**Authors:** Martin J Thomas, Gemma Hughes, Kendra Cooke, Stephanie Butler-Walley, Emma Marshall, Laura Bowyer, Simon Wathall, Jo Smith, Sarah A Lawton, June Brammar, Thomas Burnett, Chris Drake, Nadine E Foster, Gordon J Hendry, Melaine A Holden, Thomas Jaki, Royes Joseph, Anne-Maree Keenan, Jesse Kigozi, Martyn Lewis, Christian D Mallen, Hylton B Menz, Pavel Mozgunov, Edward Roddy

**Affiliations:** 1 Centre for Musculoskeletal Health Research, School of Medicine, Keele University, Keele, Staffordshire, UK; 2 Midlands Partnership University NHS Foundation Trust, Haywood Hospital, Haywood Academic Rheumatology Centre, Burslem, Staffordshire, UK; 3 Keele Clinical Trials Unit, David Weatherall Building, Keele University, Keele, Staffordshire, UK; 4 Birmingham Clinical Trials Unit, University of Birmingham, Birmingham, UK; 5 Research User Group, School of Medicine, Keele University, Keele, Staffordshire, UK; 6 Department of Mathematical Sciences, University of Bath, Bath, UK; 7 School of Medicine, University of Leeds, Leeds, UK; 8 STARS Education and Research Alliance, Surgical Treatment and Rehabilitation Service (STARS), The University of Queensland and Metro North Health, Brisbane, Queensland, Australia; 9 School of Health and Life Sciences, Glasgow Caledonian University, Glasgow, UK; 10 MRC Biostatistics Unit, University of Cambridge, Cambridge, UK; 11 Faculty of Informatics and Data Science, University of Regensburg, Regensburg, Germany; 12 NIHR Leeds Biomedical Research Centre, Leeds, UK; 13 Health Economics Unit, University of Birmingham, Birmingham, UK; 14 School of Allied Health, Human Services and Sport, La Trobe University, Melbourne, Victoria, Australia

**Keywords:** Plantar heel pain, self-management, exercise, orthoses, randomised controlled trial, adaptive design

## Abstract

**Background:**

Plantar heel pain (PHP) is the most common soft tissue foot condition and impairs mobility, physical function, ability to work, and quality of life. Systematic reviews highlight a need for high-quality randomised controlled trials (RCTs) of exercises and orthoses for PHP.

**Objectives:**

To determine the clinical and cost-effectiveness of individualised exercises and/or prefabricated foot orthoses plus self-management advice (SMA) compared to SMA alone in adults with PHP.

**Methods:**

A multi-centre four-arm two-stage adaptive parallel-group RCT with internal pilot. Up to 696 participants aged ≥18 years with PHP will be identified from general practice, physiotherapy/podiatry referrals and self-referral, and randomised 1:1:1:1 to: (1) SMA (control), (2) SMA plus individualised exercises, (3) SMA plus prefabricated foot orthoses, or (4) SMA plus individualised exercises and prefabricated foot orthoses. Outcomes will be collected by SMS text-message (weekly during weeks 1–12, monthly during months 4–12) and questionnaires at 12 weeks and 6 and 12 months. The primary outcome is change in PHP intensity (0–10 numeric rating scale) between baseline and the average over 6–12 weeks. Interim analysis when 348 participants have completed the primary outcome assessment will inform adaptation, where interventions may be dropped or the trial stopped early (for efficacy or futility). The main between-group comparison for the primary outcome will be undertaken using linear mixed modelling. Secondary outcomes will examine i) short-term pain trajectories over weeks 1–12, ii) pain at 6 and 12 months, and monthly from 3–12 months, iii) first step pain, physical function, global rating of change, pain self-efficacy, illness perceptions, ability to work, and treatment satisfaction at 12 weeks, 6 and 12 months, iv) cost-effectiveness. Patient and public partner involvement is embedded throughout.

**Discussion:**

The TREADON multi-arm multi-stage RCT will provide new evidence on the clinical and cost-effectiveness of individualised exercises and prefabricated foot orthoses for people with PHP.

**Trial registration:**

**ISRCTN** 12418153. Registration date 06 December 2022 https://doi.org/10.1186/ISRCTN12418153

## Plain Language Summary

**Aims:**

To show whether exercises and/or foot orthoses (shoe insoles) provide more pain relief for adults with plantar heel pain than a self-management advice booklet alone, and whether this is good value for money for the NHS.

**Background:**

Pain under the heel (plantar heel pain, PHP) affects 1 in 10 adults. It makes walking and everyday tasks, including work, difficult. Most people who consult general practice are given pain medication and advice, yet symptoms often continue. In our pilot and feasibility trial, exercises and foot orthoses showed promise for improving pain and function, but a larger trial is needed to confirm this.

**Methods:**

In this randomised controlled trial, up to 696 adults with PHP will be given a self-management advice booklet and allocated by chance to one of four treatments: exercises, foot orthoses, combined exercises and foot orthoses, or no additional treatment. Participants will be sent weekly text-messages or receive brief phone calls, to collect pain scores for up to 12 weeks and then monthly from 3-12 months, plus questionnaires at 3, 6 and 12 months. Half-way through the trial, only treatments that are reducing pain will continue to be offered and the trial will stop early if one treatment is clearly better.

**Patient involvement:**

Patient representatives will support the trial and helped write this summary. Five people with PHP took part in a workshop to help with trial design. For example, they helped decide how often pain should be measured. We will involve people with PHP to assist with our recruitment strategy and help us interpret the findings, develop easily understandable messages and publicise them widely.

**Dissemination:**

We will present findings at healthcare conferences, in medical journals, on relevant websites, through social media, and general practice, physiotherapy and podiatry networks. The results will be provided to participants.

## Introduction

### Background and rationale

Plantar heel pain (PHP) is a term describing several undifferentiated painful conditions affecting the plantar heel. It is now preferred to the label “plantar fasciitis”, which was used commonly in the past ^1^. It is the most prevalent soft tissue foot complaint, affecting 10% of adults, and impairs mobility, foot and physical function, and ability to work, impacting negatively on quality of life ^2– 4^. Clinical features include pain under the heel, made worse by weight-bearing, particularly after prolonged rest. Its specific cause is uncertain, although established risk factors include obesity; pronated foot posture; reduced ankle or first metatarsophalangeal joint range of motion; prolonged weight-bearing; and tightness in the gastrocnemius and soleus muscles, plantar fascia, and Achilles tendon ^2, 5^. Weakness of the gastrocnemius, soleus, and intrinsic foot muscles has also been implicated ^6^.

PHP is commonly stated to be a benign, self-limiting condition that, for most people, resolves within one year ^7^. However, symptoms can become chronic and persistent: in some studies almost half report persistent symptoms after ten years, leading to impaired quality of life, physical inactivity and weight gain ^4, 8^. A National Institute for Health and Care Excellence (NICE) Clinical Knowledge Summary recommends initial treatment with analgesia and advice regarding rest, footwear, heel pads, weight loss and stretching exercises ^7^. Referral to a podiatrist or physiotherapist is advised if mild symptoms persist beyond a few months despite conservative treatment or if symptoms are severe. In the Netherlands and Australia, the most frequent strategies employed by General Practitioners (GPs) to manage PHP are referral to a podiatrist (12–20%), watchful waiting (18–32%), non-steroidal anti-inflammatory drugs (NSAIDs) (20–23%), and advice to wear insoles (16%) ^9, 10^. Qualitative studies show that the expectations and needs of people with PHP are often unmet ^11^.

Healthcare professionals commonly use foot orthoses and/or lower limb exercises to treat patients with PHP ^12^. Foot orthoses are insole devices designed to optimise foot loading distribution and adjust medial longitudinal arch function through control of specific foot motion ^13^. They reduce rearfoot pronation in a dose-dependent manner, addressing planus foot posture (flat feet) and excessive foot pronation (eversion) associated with PHP, and increase foot-to-surface contact area, lowering plantar heel pressures and tensile stresses at the calcaneal-plantar fascia junction during loading to provide symptomatic relief from PHP ^13– 17^. Exercises, including stretching the Achilles tendon and plantar fascia and strengthening of the intrinsic muscles of the foot and lower limb muscles (e.g. hamstrings, quadriceps and triceps surae), aim to improve movement and restore normal foot loading ^18– 20^. Tightness and weakness in the plantar fascia, Achilles tendon and foot muscles are therefore viable therapeutic targets for individualised exercises in the treatment of PHP. However, orthoses and exercises are infrequently used in general practice to treat this common and disabling condition despite many people having persistent problems ^9, 11^.

The evidence-base to inform clinical decisions about treatments is limited and often of poor quality ^21– 23^. Many published randomised controlled trials (RCTs) are limited by small sample sizes, short-term follow-up and poor methodological quality, and there is a need for larger trials incorporating longer follow-up, more robust methods, and higher reporting standards ^21– 23^. Two systematic reviews of foot orthoses compared with sham orthoses for PHP published in 2018 reached different conclusions despite pooling data from the same three trials: one found moderate-quality evidence that foot orthoses are effective at reducing pain in the medium term (7–12 weeks) but had no effect in the short term or on function ^21^, whereas the other found that foot orthoses were ineffective ^22^. This difference has been attributed to the reviews extracting outcome data for different measures of foot pain ^24^. Our previous network meta-analysis found that exercises alone did not reduce pain or improve function in patients with PHP in the short or medium term, but improved function at 12 months compared with placebo/sham interventions, based on only two small RCTs (<50 participants each) ^23^. No treatments commonly used for the management of PHP (including orthoses and exercises) were significantly better than any other for pain and function. Placebo/sham interventions and NSAIDs were the least effective, suggesting that current recommendations for first-line management of PHP with analgesics, NSAIDs and watchful waiting may be suboptimal. We found only one RCT which examined the effectiveness of combining orthoses and exercises ^25^. Prefabricated orthoses plus stretching exercises were more effective than stretching exercises alone although the trial was small (42–51 participants per arm), follow-up was short (8 weeks) and risk of bias was high.

Subsequently, one small RCT (n=95) reported greater improvement in pain with usual podiatry care plus physical therapy than with usual podiatry care alone at one year, but there was no difference in physical function ^26^. A larger RCT (n=185) concluded that referral to a podiatrist for custom-made insoles does not lead to better outcomes compared with usual GP care ^27^. However, usual GP care within the trial was more intensive than in routine practice with 41% of participants receiving biomechanical interventions such as heel cups and 15% corticosteroid injection ^27, 28^. Other recent small-to-moderate sized trials have investigated foot orthoses versus corticosteroid injection (n=103) ^29^, exercise versus corticosteroid injection plus exercise (n=180) ^30^, insoles versus sham insoles adapted for flip-flop sandals (n=80) ^31^, and radial extracorporeal shock wave therapy versus sham radial extracorporeal shock wave therapy versus exercise versus advice plus customised foot orthoses (n=200) ^32^.

There is currently no evidence of cost-effectiveness of exercise programmes for PHP, and evidence for orthoses is limited to one study which showed that prefabricated orthoses were similarly effective to customised orthoses but significantly less expensive ^33^. Informed by our successful feasibility and pilot trial, which reported no serious adverse events ^34^, the TReatments of Exercise AnD Orthotics for plaNtar heel pain (TREADON) trial will investigate the clinical and cost-effectiveness of individualised exercises and/or pre-fabricated foot orthoses plus self-management advice (SMA) compared with SMA alone for adults with PHP in primary care over the medium and long term. Such a trial is needed to support clinical decision-making and inform National Health Service (NHS) policy and commissioning pathways for this patient group who experience considerable pain and functional limitation but are often overlooked in terms of treatment options that are available but only accessed by a few ^9, 10^.

## Protocol

The full current version of the protocol (Version 3.1, 07 October 2024, at the time of submission) can be viewed on the funder website: https://fundingawards.nihr.ac.uk/award/NIHR131638. Details of previous protocol versions and amendments are presented in Table 1.

**Table 1.** Previous protocol versions and amendments.

Version	Issue date	Reasons for amendments; additional changes
1.0	01 September 2022	Version originally submitted for NHS Health Research Authority (HRA)/ethics committee approval.
2.0	08 December 2022	Version approved by NHS HRA/ethics committee. • Updated that electronic follow-up questionnaires will be sent via a link in an email. • Clarified what is in a full trial information pack for patients indicating postal or electronic contact.
3.0	02 April 2024	Substantial amendment (SA01): • Flexibility added to time for sending clinician appointment invitation reminders allowing local decisions on how best to manage participants, agreed with the TREADON patient advisory group. Changed from being sent at 4 weeks to being sent between 2 and 4 weeks. Also updated in flow charts version 2.0. • Updates to subgroup analyses description and procedures relating to missing or spurious data. • Addition of detail relating to estimands to be used in trial analysis. • Clarity on processes for following up eligible people for consent and baseline data. • For online participants; sending a sample consent form and baseline questionnaire prior to the eligibility call, which is then followed up by a second email asking them to complete these documents. • Treatment Diary Reminder email – to be sent as a reminder for instances where this is the only known method of contact. • Updates to list of abbreviations, staff titles and email address. • Clarified that up to 6 appointments refers to 1 initial and up to 5 follow up appointments. Also, that the intervention training may involve private study such as watching pre -recorded videos. • Updates to the wording relating to GP searches to reflect IT system differences and limitations across UK nations. • Clarified that those on EMIS PCS / Cegedim (Vision) will use a smaller Read code list. • Corrected wording around process of notifying online participants of treatment allocation – Via email or by post as opposed to through a link URL. • Removal of term 'working days' to just use 'days' and abbreviation of Participant Information Leaflet to PIL. Removed ‘Preferred rolling monthly method’ from the first figure in the extended data file. This is included in trial flow charts to make the version 2.0.
3.1	07 October 2024	In line with the eligibility CRF, updates made to the protocol to: • Clarify inclusion criterion is self-reported localised pain under the heel which is worse when standing after rest and after prolonged weight-bearing (i.e. change word ‘worst’ to ‘worse’). • Add examples of inflammatory arthritis to exclusion criteria. • Removed examples of allergies to common orthotic device materials. • Specified that ‘unable to attend for treatment’ means ‘Unwilling or unable to participate with the interventions or attend clinics for treatment’. • Clarified that when 1 year has passed since the first invitation date for Method 2, GP practices may conduct a further clinical system search to identify adults aged 18 years and over that have a SNOMED/Read coded consultation for foot/ankle pain in the preceding year, excluding any patients that were previously invited. • Removed ‘Delay in treatment initiation’ from estimand to correspond with an update to the Data Analysis Plan.

### Objectives

The overall aim of the trial is to compare the additional benefit of individualised exercises and/or prefabricated foot orthoses versus a SMA booklet alone to treat adults with PHP in primary care.

***Primary objective/research question***

In adults with PHP, does a SMA booklet combined with individualised exercises and/or prefabricated foot orthoses lead to greater improvement in pain in the medium term (average pain over 6–12 weeks of follow-up) than a SMA booklet alone?

***Secondary objectives***

To compare, in adults with PHP:

•   The effect of a SMA booklet combined with individualised exercises and/or prefabricated foot orthoses with a SMA booklet alone on;

1. short-term pain trajectories over weeks 1 to 12, including individual weekly comparisons,
2. pain at 6 and 12 months, and monthly from 3 months to month 12,
3. first step pain, physical function, patient global rating of change, pain self-efficacy, illness perceptions, ability to work, and treatment satisfaction at 12 weeks, 6 and 12 months.

•   The cost-effectiveness of a SMA booklet combined with individualised exercises and/or prefabricated foot orthoses with a SMA booklet alone.

***Outcome measures/endpoints***

Informed by the TREADON pilot and feasibility trial, the primary outcome is a pain intensity change score using a 0–10 numeric rating scale (NRS). The end points are defined as:

•   Primary end point at 6–12 weeks for clinical effectiveness and at 12 months for cost-effectiveness analysis.

***Primary endpoint/outcome***

The primary outcome for clinical effectiveness is change in PHP intensity (0–10 NRS) between baseline and the average rating over weeks 6 to 12, collected by weekly Short Message Service (SMS) text-message or brief phone call between weeks 1–12.

***Secondary endpoints/outcomes***

Secondary outcomes will comprise PHP intensity score (0–10 NRS), first step pain (0–10 NRS), Foot Function Index ^35^, (pain, disability, activity restriction subscales, and overall), patient global rating of change, pain self-efficacy questionnaire ^36^, brief illness perceptions questionnaire ^37^, quality of life (EuroQol 5-Dimension 5-level instrument (EQ5D-5L)) ^38^ work loss and presenteeism ^39^, self-reported healthcare use for PHP (NHS and private), self-reported treatment adherence, treatment credibility, satisfaction with care, and adverse events.

**Adverse events:** Trial-related adverse events (for example skin irritation from orthoses, muscle soreness from exercise) will be captured through case report forms (CRF) completed by TREADON physiotherapists/podiatrists, and direct contact between the Keele Clinical Trials Unit (CTU) and the participant, their TREADON physiotherapist/podiatrist, GP or site Principal Investigator (PI). Participants randomised to the clinician-supported intervention arms will record adverse events in a weekly diary for the 12-week intervention period.

Trial safety reporting procedures will ensure any unexpected serious adverse events which are deemed related to the trial will be reported to the Research Ethics Committee and Sponsor.

All Serious Adverse Events either confirmed or suspected to be related to the trial procedures will be reviewed by the independent Data Monitoring Committee (DMC) and reported to the Trial Steering Committee (TSC). The TSC reports directly to the funder.

***Estimand***

The primary estimand of interest (corresponding to a treatment policy strategy, as suggested in the International Conference of Harmonisation E9 (R1) addendum on estimands and sensitivity analysis in clinical trials) ^40^ is the mean difference in the change in PHP intensity (0-10 NRS) between the baseline and the average rating over weeks 6 to 12, in eligible and randomised adults with PHP intended to be treated with individualised exercises and/or prefabricated foot orthoses in addition to a SMA booklet compared with SMA booklet alone, regardless of treatment adherence, initiation of other treatment, adverse events, intervention fidelity, or other protocol deviations such as treatment switching and subsequent ineligibility (Estimand 1). Thus, a treatment policy approach (the data will be collected and analysed regardless of whether the intercurrent event occurs) is applied for the intercurrent events (intercurrent events occur after randomisation and affect the interpretation of the trial outcome).

In addition to the primary estimand, an alternative estimand (Estimand 2) targeting a different clinical question of interest — treatment effect in the absence of treatment non-compliance and other protocol violations — will be explored in a supplementary analysis where the treatment non-compliance and other protocol violations will be treated under a hypothetical strategy (any data collected after the intercurrent event, which is affected by the intercurrent event, will be treated as missing).

Full details of Estimands 1 and 2 will be provided in the TREADON trial's Data Analysis Plan.

## Methods

**Trial registration: ISRCTN** 12418153. Registration date 06 December 2022 https://doi.org/10.1186/ISRCTN12418153

### Patient and Public Involvement and Engagement

Patient representatives were involved in our pilot and feasibility trial, informing development of the trial interventions, and will support every stage of the main trial. Our lay co-investigator (JB) helped write the Plain English Summary and is a member of the Trial Management Group. Five people with PHP took part in a workshop to help design the main trial. They recommended inclusion of Participant Identification Method 5 (self-referral from the community), helped us decide how often pain should be measured, and advised on how to optimise adherence to exercise. We will also involve people with PHP to assist with our recruitment strategy and help us to interpret the trial findings, develop easily understandable messages to explain the findings and publicise the findings widely. Two people with PHP will sit on the TSC.

### Ethics and consent statement

Ethical approval was obtained from West of Scotland Research Ethics Service (reference number: 22/WS/0165) on 22 November 2022. Those who wish to take part in the trial will be asked to sign and date the consent form included in their baseline pack and return to the research team either in electronic-consent online format or in the pre-paid envelope provided to those who opt for paper-based involvement.

### Trial design

Multi-centre, randomised, parallel group, four-arm two-stage adaptive trial, with 6-month internal pilot.

***Interventions***

The duration of intervention regardless of group allocation will be 12 weeks. Participants will be asked not to use other types of treatments, other than medication that their GP has provided (where relevant), during the intervention period if possible; however, any additional healthcare and self-care use will be recorded in the 12-week follow-up questionnaire, 6-month follow-up questionnaire and 12-month follow-up questionnaire.

***SMA booklet (control arm)***

Participants randomised to receive SMA only will be mailed a SMA booklet, based on the Versus Arthritis leaflets on plantar fasciitis ^41^ and foot and ankle pain ^42^, supplemented with specific advice and information including a small number of stretching exercises and self-help messages about pain relief, footwear, rest and weight loss, consistent with best practice guidance ^43^. The booklet describes five stretching exercises for the plantar fascia and Achilles tendon to be performed twice per day ^41^, without instructions for individualisation, progression or supervision of exercises (see extended data).

***SMA booklet plus individualised exercises (SMA-exercises)***

**Figure 1.**
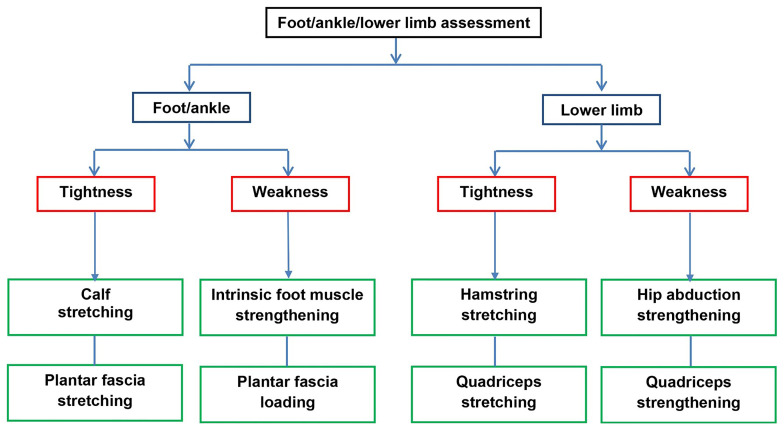
Summary flow chart of exercise prescription.

Participants randomised to SMA-exercises will be given the SMA booklet at the initial treatment appointment. The treating physiotherapist or podiatrist will assess foot posture and function to determine the exercise type and dose. At the discretion of the clinician, a more generic lower limb assessment of alignment and function can also be undertaken, enabling strengthening and stretching for hip abductors, quadriceps and hamstrings to be prescribed if deemed to be important as part of the overall PHP treatment. Exercise selection will be informed by the level of clinically-observed muscle tightness, weakness and functional limitation (see Figure 1). Exercise dose will be individualised and progressed, based on assessment findings and informed by current exercise guidelines ^44^. The exercises are drawn from best available evidence ^5, 18– 20, 45, 46^ and discussion with clinicians during workshops prior to our pilot and feasibility trial. Exercises include foot-specific stretches/exercises targeting the plantar fascia, intrinsic foot muscles, Achilles tendon, key ankle-related muscle groups such as soleus and gastrocnemius, and other muscle groups in the lower limb identified as targets within the assessment. Participants will be taught how to perform and progress these exercises and given an individualised and detailed exercise sheet (online or paper) describing the regimen and showing photographs of the exercises. The exercise sheets provided will be trial-specific exercises selected by the clinician and comprising photographs and written instruction completed by the clinician (see extended data). Participants will be offered up to 6 treatment sessions (1 initial and up to 5 follow ups) over 12 weeks at the discretion of the treating clinician (face-to-face appointments prioritised if possible, or virtual, telephone, if required).

Exercise programme modification will be based on a subjective and/or objective re-assessment and include either:

1. progression of prescribed exercises if tolerated with minimal pain and discomfort,
2. maintenance if tolerated but some moderate pain and discomfort, or
3. reduction in frequency, duration, intensity or modification of exercise type if not tolerated or adhered to.

The exercise programme will be supervised during each treatment appointment and may be progressed at subsequent treatment appointments according to observed changes in presentation, modelled on successful exercise interventions in our earlier trials ^47, 48^. A record of the exercise prescription will be recorded on the Intervention Details CRF for each treatment appointment. Adherence will be encouraged by provision of written individualised exercise sheets (online or paper) and use of a paper-based diary to discuss with the clinician during subsequent treatment appointments. The diary will be collected at 12 weeks.

***SMA booklet plus prefabricated foot orthoses (SMA-orthoses)***

**Figure 2.**
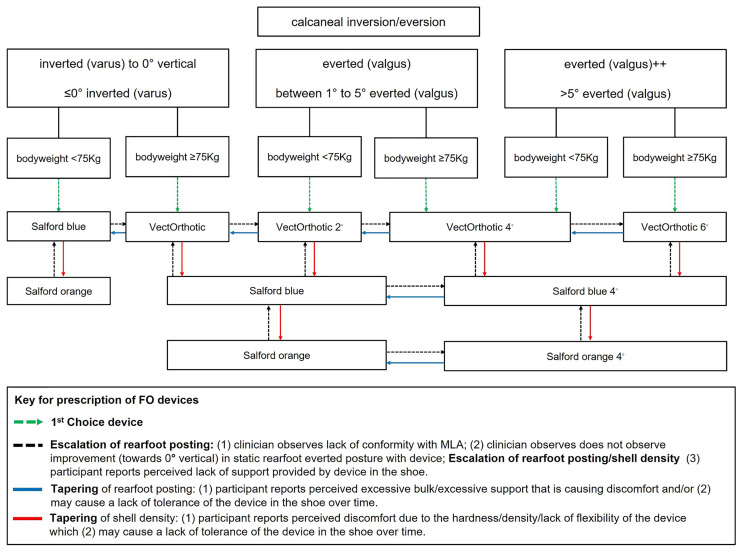
Summary flow chart of orthoses prescription.

Participants randomised to SMA-orthoses will be given the SMA booklet at the initial treatment appointment. The treating physiotherapist or podiatrist will assess foot posture using the Foot Posture Index-6 (FPI-6) ^49^ and select the appropriate orthotic device according to the degree of static rearfoot eversion and body weight (see Figure 2 for the foot orthoses and body weight algorithm). These data will be recorded on the CRF by the treating clinician at each appointment. If deemed clinically relevant to PHP, this can include assessment of hip position (anteversion/retroversion) and knee position (genu varus/valgus).

**Protocol for prescription of prefabricated foot orthoses**

The assessment protocol for the prescription of foot orthoses includes two key components to guide the physiotherapist/podiatrist towards the most appropriate foot orthosis prescription.

1.   A main driver in influencing the rearfoot posting component of the foot orthosis prescription will be the rearfoot posture component of the FPI-6 ^49^. This will be assessed by the physiotherapist/podiatrist when the participant is in a relaxed stance position. The physiotherapist/podiatrist will observe whether the calcaneus is inverted, vertical, everted, or highly everted (see Figure 2).

2.   Assessment of body weight will also influence the selection of the appropriate orthotic device (greater body weight resulting in selection of a device with a higher material density).

3.   Each foot orthotic device will be fitted according to the size of the participant’s foot using the orthotic device shells of various sizes.

4.   Clinicians will select the appropriate first choice device with appropriate rearfoot medial posting dose (see Figure 2) in place and assess the participant for correct size of orthotic device (weight-bearing and non-weight-bearing fit-to-foot).

5.   Clinicians will then check the fit of the orthotic device to the shoe (fit-to-shoe).

6.   Tolerance will be evaluated by asking the participant if they are happy with the comfort and fit of their orthotic device (tolerance). If the participant is not happy with comfort or fit, the clinician may choose to taper the shell density and/or the dose of rearfoot posting.

7.   Foot orthoses should be prescribed for both feet according to the intervention protocol, which is driven by participants' foot posture and bodyweight, regardless of whether the heel pain presentation is unilateral or bilateral.

**Foot orthoses choice**

In the development of our pilot and feasibility trial ^34^, our patient and clinician advisory group identified desirable characteristics of an orthotic intervention protocol including:

1. an element of patient choice between different devices based on comfort and fit
2. scope for device adjustment/tailoring by the clinician to provide the desirable level of rearfoot posture/functional control for individual symptoms.

As a result, we developed a pragmatic foot orthosis intervention algorithm, which includes the following devices:

- Vectorthotic® (firm density shell), (with CE marking; Healthy Step; Ashton-under-Lyme, UK)
- Salfordinsole™ Firm (medium to firm density shell), (with CE marking; Salfordinsole Health Care Ltd; Nuneaton, UK)
- Salfordinsole Flex (low-medium density shell).

These devices are prefabricated and modifiable with the use of ‘click-in’ or adhesive additions (medial rearfoot posts) which can be used to change the level of pronatory control, as well as patient comfort and therefore potentially influence adherence. A range of shell material densities allows for a more supportive orthotic for participants with a higher bodyweight ^50^. At present, there is little evidence to suggest that one brand/type of foot orthoses is more effective than another for the management of PHP ^21, 51^. Participants will be instructed how to fit the device and advised to wear it for one hour per day, gradually increasing by one hour per day up to at least four hours per day, and given a detailed orthosis information sheet. Participants will be offered up to 6 treatment sessions (1 initial and up to 5 follow ups) over 12 weeks at the discretion of the treating clinician (face-to-face appointments prioritised if possible, or virtual, telephone, if required). The orthosis can be altered during subsequent consultations according to participants’ self-reported tolerance or clinical presentation. Adherence will be encouraged by a diary to capture use and facilitate discussion with the clinician. The diary will be collected at week 12. The foot orthosis may be changed or altered during subsequent clinical treatment appointments according to participants’ self-report of tolerance or clinical presentation.

**Foot orthoses details**

*Materials*: Vectorthotic devices - semi-rigid polypropylene shell with a closed cell polyethylene cover and polypropylene 2°, 4° and 6° rearfoot posts, Salfordinsole shells and 4° rearfoot posts, both Sure Step-Control™ thermoplastic elastomer material.

*Supplier details:* Participants randomised to receive foot orthoses will receive a trial orthotic device. These will be provided to participants free of charge in accordance with the Supply Instructions provided in the Investigator Site File.

*Storage and consignment stock:* Orthotic devices will be stored in accordance with manufacturer’s recommendations and as outlined in the supply instructions provided in the trial information. A consignment stock of orthoses will be sourced and purchased by a member of the research team and delivered to each treatment site. This will ensure that participants can be provided with the appropriate orthoses, as required. The PI at each site is responsible for consignment stock monitoring and replenishment.

***SMA booklet plus individualised exercises and prefabricated foot orthoses (SMA-combined)***

In addition to the SMA booklet provided at the initial treatment appointment, participants randomised to SMA-combined will be offered both individualised exercises and prefabricated foot orthoses interventions as described above and delivered over up to 6 treatment sessions (1 initial and up to 5 follow ups), over 12 weeks, by a physiotherapist or podiatrist.

***Treatment appointment procedures***

For all three clinician-supported intervention arms (SMA-exercises; SMA-orthoses; SMA-combined), participants who do not attend (DNA), or who are unable to attend (UTA) their physiotherapist/podiatrist appointment will be offered further appointments for the duration of treatment and CRFs will be completed to note the DNA/cancellation. Clinicians will be trained to deliver both exercise and orthosis interventions and will be notified which arm the participant has been randomised to in advance of their first treatment session. Clinicians will be asked to complete a treatment CRF for each participant at each appointment. Table 2 summarises each intervention arm.

**Table 2.** Summary table of interventions.

Self-management advice	Self-management advice *plus* individualised exercises	Self-management advice *plus* prefabricated foot orthoses	Self-management *plus* individualised exercises and prefabricated foot orthoses
Advice and information booklet	Advice and information booklet Up to 6 sessions (1 initial and up to 5 follow ups) over 12-week treatment period Individually tailored and written exercise programme Focus on foot/ankle specific stretching and strengthening +/- lower limb muscle groups Individualised exercise programme that is progressed Clinical supervision of exercise programme Exercise diary	Advice and information booklet Up to 6 sessions (1 initial and up to 5 follow ups) over 12-week treatment period Individually tailored pre-fabricated foot orthotic device Device adjustment/ tailoring as required Clinical supervision of orthotic device use/tolerance Orthosis diary	Advice and information booklet Up to 6 sessions (1 initial and up to 5 follow ups) over 12-week treatment period Individually tailored and written exercise programme Focus on foot/ankle specific stretching and strengthening +/- lower limb muscle groups Individualised exercise programme that is progressed Clinical supervision of exercise programme Individually tailored pre-fabricated foot orthotic device Device adjustment/tailoring as required Clinical supervision of orthotic device use/tolerance Exercise & orthosis diary

***Trial training***

Treatment packages for those participants randomised to interventions which include exercises or foot orthoses, will be delivered by participating physiotherapy and podiatry sites. Participating services will be those who have agreed to participate, have local management approval, have been trained in trial-specific interventions and procedures, and are accessible to patients registered at the general practices participating in the trial. A research clinician (e.g. physiotherapist, podiatrist, nurse) will be trained to perform telephone screening of potential participants, confirm eligibility and obtain consent. Trial administrators will also be trained to support site processes (e.g. booking participants into treatment appointments), as required.

**Physiotherapists and podiatrists:** Treating physiotherapists and podiatrists (at any level of post-registration experience) will participate in a two-day training workshop with the research team prior to the start of recruitment (or as a hybrid remote and face-to-face delivery with additional private study [watching pre-recorded presentations and completing self-directed activities] if this flexibility is more efficient for clinical service engagement at treatment sites, or remote delivery only if the COVID-19 pandemic or any other similar situation requires). The workshop(s) will be based on the training provided in our pilot and feasibility trial. The focus of the training will be on carrying out standardised assessment, delivery of the exercises and orthosis interventions in line with the agreed protocols (including remote delivery if the COVID-19 pandemic requires), and supporting participants to adhere to the interventions, with particular emphasis on the SMA-exercises arm. The training also will cover completion of all trial paperwork requirements including CRFs to assess intervention fidelity, Good Clinical Practice (GCP) as applicable to research, maintenance of the site file and trial records, and reporting of serious adverse events, serious breaches, adverse events and protocol non-compliance.

Based on lessons learnt from the TREADON pilot and feasibility trial ^34^, in the clinician training programme, we will emphasise the importance of supervision and progression of exercises by utilising the available number of treatment sessions and strategies to improve adherence to exercises such as encouraging completion and review of exercise diaries. Throughout the training programme, we will emphasise the key characteristics of the therapeutic alliance that facilitates exercise adherence, including, agreement on goals and tasks (goal setting), clear communication, a sense of connectedness, positive feedback, genuine interest, individualised care plans (including exploring specific barriers to exercise adherence), trust in the clinician, and feeling empowered ^52, 53^.

The training will be supplemented by a comprehensive manual, providing clear treatment protocols and guidance for completing trial paperwork. CRFs completed by clinicians to capture data on treatment delivery will be regularly audited against the treatment protocol to examine if they are being delivered in accordance with the protocol. Individual feedback will be given to clinicians if required. Refresher training will be offered where needed.

### Trial setting

Participants will be recruited from general practices, NHS physiotherapy and podiatry services, and from self-referral direct to the trial research team from people living in the community following an awareness raising campaign within England and Scotland. Interventions will be delivered in TREADON-specific treatment sessions that will be integrated into routine NHS physiotherapy and podiatry services. This will ensure sampling from a heterogeneous population spanning a range of area-level deprivation/affluence and a mix of urban, semi-rural and rural areas. Participating physiotherapy and podiatry services and their geographical locations information can be found on the trial website ( https:///www.keele.ac.uk/treadon/cliniclocations/).

### Eligibility criteria

The target population is adults aged 18 years and over with PHP. Our inclusion/exclusion criteria are deliberately broad ( Table 3). Only those who have symptoms unlikely to be attributable to PHP, current/recent experience of the interventions in the trial, or for whom the interventions would be contraindicated/inappropriate will be excluded. There are no exclusions based on language (however the SMA booklet is only available in English) as we will utilise local interpreter services (e.g. NHS, local authority) at all stages of screening, consent, treatment and data collection to ensure the trial is accessible to non-English speaking populations.

**Table 3.** Eligibility criteria.

Inclusion criteria
Adults aged 18 years and over
Self-reported localised pain under the heel which is worse when standing after rest and after prolonged weight-bearing
Symptom duration at least 4 weeks with pain ≥2 (0-10 Numeric Rating Scale [NRS])
Access to a mobile phone that can send/receive SMS text-messages or a landline telephone
Able and willing to participate and provide informed consent
Exclusion criteria
Inflammatory arthritis (e.g. rheumatoid arthritis, ankylosing spondylitis, reactive arthritis, psoriatic arthritis), systemic lupus erythematosus, gout, fibromyalgia
Heel pain of neurologic cause e.g. tarsal tunnel, entrapment neuropathy, radiculopathy
Serious pathologies requiring urgent medical attention (e.g. trauma, tumour, infection)
Current treatment or treatment in the last 3 months by a physiotherapist or podiatrist for plantar heel pain, or currently using a contoured foot orthosis
Previous surgery or awaiting surgery on the affected foot
Corticosteroid injection into the affected foot in the last 3 months
Extracorporeal shockwave therapy to the affected foot in the last 3 months
Unlikely or unwilling to participate with the interventions or attend clinics for treatment
Unlikely to tolerate the interventions (e.g. allergies to common orthotic device materials)

### Trial procedures

***Recruitment***

**Patient identification**

Potential participants will be identified based on the three methods that were employed successfully in our pilot and feasibility trial: 1) general practice consultation, 2) retrospective review of general practice medical records, 3) population survey ^34^. In addition, and to further optimise recruitment, the following methods will also be employed, 4) screening referrals to NHS physiotherapy and podiatry services and, on the advice of our Patient and Public Involvement and Engagement (PPIE) group, 5) identify participants through community advertising and self-referral (see extended data). All recruitment arms will proceed in parallel until the required sample size is reached.

**Method 1 – Rolling monthly identification - General practice consultation: Foot/ankle pain consulters**

***Clinical System Protocol***

Potential participants will be identified through routine primary care consultation (face-to-face or telephone) about PHP at their general practice. Where possible, a trial-specific general practice clinical system protocol will automatically identify, screen and code potential participants aged 18 years and over. The protocol will trigger when specific Systemised Nomenclature of Medicine (SNOMED) codes or Read codes are entered into a general practice consultation; these codes are based on foot/ankle pain related diagnostic or symptomatic Read or SNOMED codes that were successfully used to identify adults with PHP in our pilot and feasibility trial ^34^. As in our pilot and feasibility trial, a broad range of symptom codes will be used because foot/ankle consultations are often not coded with specific diagnostic labels such as PHP ^54^. The protocol will automatically exclude those patients with potentially serious pathology (e.g. inflammatory arthritis, as listed in the exclusion criteria) and potentially vulnerable patients. The clinician triggering the protocol will be asked to confirm if the patient is suffering from PHP and therefore eligible to receive further information. A clinical system search will be manually run approximately every 4 weeks to identify these eligible patients.

***Clinical System Search***

Practices which use the Clinical Systems EMIS PCS/Cegedim (vision), do not have the required functionality to create a Clinical System protocol suitable for the trial. In addition, some practices may decline the use of a system protocol. In these practices, a trial-specific clinical system search will be run to identify patients who have had a qualifying foot/ankle consultation in the last four weeks. For consistency, for those practices using the EMIS PCS and SystmOne systems, the search will utilise the exact same SNOMED code list that the protocol uses to identify (trigger) and for the automated exclusions. Practices using the EMIS PCS/Cegedim (Vision) systems, due to system limitations and using Read code terminology, will use a shorter more focused list of codes for inclusion and exclusion. A practice clinician will then perform a manual screen of this list to confirm if plantar heel pain is present.

***Patient contact***

Approximately every four weeks, the general practice will send potentially eligible patients identified in the last four weeks, an SMS text-message containing a link Unique Resource Locator (URL) to the online screening survey pack (including the survey participant information leaflet (PIL), online consent to contact reply form and the screening survey), with a reminder SMS text-message to be sent within seven days after the initial message.

Patients who do not have an active mobile number, or who have opted out of SMS contact from their general practice, will be sent the screening survey pack by post (an invitation letter from their GP, survey PIL, postal consent to contact reply form and screening survey with a pre-paid return envelope) via Docmail, which is a standards-compliant hybrid mail service, providing document management and ISO 27001 secure mailings.

The screening survey will ask respondents to indicate the location of pain on a validated foot manikin ^55, 56^ (© The University of Manchester 2000. All rights reserved) and complete questions regarding foot pain and demographic details. For those completing the foot manikin online, the participant will select the location of pain on the foot manikin. For those completing a paper survey, the participant will shade directly the location of pain on the manikin, which will be read by overlaying a reliable coding template previously used in our foot pain research ^56^.

Non-responders will be sent a reminder approximately two to four weeks after the initial invite. Pilot and feasibility trial recruitment rate was 6/10,000 practice population ^34^.

**Method 2 – Retrospective (1 year) general practice search: Foot/ankle pain consulters**

The general practice will run a trial-specific clinical system search to identify adults aged 18 years and over who have a SNOMED/Read coded consultation for foot/ankle pain in the preceding year. The clinical criteria of the search for inclusion and exclusion for the EMIS PCS and SystmOne systems will utilise the same SNOMED code lists used in Method 1. General practices using the EMIS PCS/Cegedim (Vision) systems, due to system limitations and using Read code terminology, will use a shorter more focused list of codes for inclusion and exclusion.

The general practice will then contact eligible screened patients via the SMS and Docmail methods outlined in Method 1 above.

When one year has passed since the first initiation date, general practices may conduct a further clinical system search to identify adults aged 18 years and over that have a SNOMED/Read coded consultation for foot/ankle pain in the preceding year. This search will exclude any patients that were previously invited.

Non-responders will be sent a reminder approximately two to four weeks after the initial invite. Potential participants who appear eligible and who consent to contact will be sent a full trial information pack either online or postally. Pilot and feasibility trial recruitment rate was 2.6/10,000 practice population ^34^.

**Method 3 – General practice population survey**

All adults registered at selected general practices will be contacted to identify those who had heel pain but had not consulted in the preceding year. Our data indicate that only 43% of people with PHP consult their GP ^2^. The general practice will run a trial-specific clinical system search to identify all registered adults (≥18 years of age and exclude potentially vulnerable patients), and GPs/primary care clinicians will screen lists to exclude potentially vulnerable patients. Patients will be contacted, and non-responders followed up, as described in Method 1 above. Pilot and feasibility trial recruitment rate was 27/10,000 practice population ^34^.

**Method 4 - Physiotherapy and podiatry referrals**

To capture potential participants who have either been referred from general practice or self-referred to physiotherapy/podiatry, the NHS trust/Health Board will screen referrals against eligibility criteria to identify adults with PHP. Where IT systems allow, identified patients will be sent an SMS text-message from the physiotherapy/podiatry service providing a link URL to the online screening survey pack (including the survey PIL, online consent to contact reply form and the screening survey). Patients who are unable or unwilling to complete this online will be able to opt for postal completion. Where IT systems do not have SMS function or where patients do not have an active mobile number, patients will be sent the postal screening survey pack from the physiotherapy/podiatry service or via Docmail. Non-responders will be sent a reminder approximately two-four weeks after the initial invite.

**Method 5 – Self-referral from the community**

Our PPIE group told us that not all individuals with PHP will consult healthcare professionals about it. Therefore, adults with PHP will be able to self-refer into the trial. Local communities around each participating NHS service will be provided with information about the trial via an awareness raising campaign. Informed by previously successful awareness raising campaigns in the EASE Back feasibility and pilot trial ^57^ and PROP OA trial ^58^ and PPIE feedback, information directing individuals to the research team’s online/telephone contact details will be shared via social media (Facebook and Twitter/X), a trial website, local radio and newspapers, and flyers, posters in general practices, physiotherapy and podiatry practices, and community settings. Where possible, automated check-in screens for patients attending general practices, and waiting room display screens, will display information about the trial. Potential participants who register their interest via the trial website will be directed to the online screening survey pack (including the survey PIL, online consent to contact reply form and the screening survey).

Those contacting Keele CTU directly will be given the option to complete the screening survey either online or via post. Those wishing to complete online will be provided with a link URL to the online screening survey pack. Patients who are unable or unwilling to complete this online will be able to opt for postal completion. Non-responders will be sent a reminder approximately two to four weeks after the initial survey invite. Patients opting to complete the survey online will be advised in the PIL that they can stop completing the questionnaire at any time point, without giving a reason, but that answers entered up to that point will be kept as a partially completed survey. Patients can opt out of this by contacting the trial team.

***Duplicate invitations***

All communications to the patients identified via methods 1–4 will be from the general practice or NHS trust/Health Board participating services. Whereby a patient is identified by their general practice via methods 1, 2 or 3, systematic searches will be conducted to ensure that potentially eligible participants will not receive duplicate invitations. Invitation correspondence with potential participants will include a statement that we cannot completely eliminate the possibility of duplicate invitation.

***Full trial information pack***

Participants will be asked to indicate their preferred method of contact (for trial information and questionnaires), either as post or electronically, on their ‘consent-to-contact’ form. To facilitate online methods, email address and mobile number will be requested from those who prefer electronic contact.

For patients indicating preference for postal contact, the full trial information pack (pre-coded with a participant trial ID number) will be sent by post, from Keele CTU and will include:

- a cover letter and/or invitation to consider participation in the trial
- participant information leaflet (PIL)
- trial consent form
- baseline questionnaire (pre-treatment questionnaire)
- pre-paid return envelope (as required).

For patients indicating preference for electronic contact, an email will be sent from Keele CTU which invites then to consider participation in the trial but instructs them not to complete the attached documents, advising that, if eligible and willing to participate, a second email will follow the telephone screening call with a link to the online version of these documents:

- PIL
- a sample trial consent form
- a sample baseline questionnaire (pre-treatment questionnaire).

This will allow potentially eligible participants to consider the trial in detail in their own time.

***Telephone screening***

Potential participants sent a full trial information pack by any of the five recruitment methods will be called by a clinical researcher, within two to five working days after sending the full trial information pack, to explain the trial, confirm eligibility and facilitate consent to participate. Up to five attempts will be made to contact the patient, including at different times of the day.

The completed trial consent form and baseline questionnaire will be returned to Keele CTU online or by post.

Those patients who have verbally consented to take part in the trial will be asked to complete the baseline questionnaire included in the trial pack and return it to the research team along with a signed and dated consent form in the pre-paid envelope provided (or equivalent online correspondence). On receipt at Keele CTU, the authorised trial administrator will check it for completeness before the participant proceeds to the randomisation stage. Online correspondence checking will be performed by automated processes. Participants who are eligible but have not returned their consent form and/or baseline questionnaire will be contacted by phone after seven days of the original consent phone call date, to remind them to return their required correspondence. Where phone contact is not made or if after a further seven days from the reminder phone call, the patient still has not returned their required correspondence, a letter will be sent to remind them to return their questionnaire.

### Consent

***Consent to full trial participation***

Those who wish to take part in the trial will be asked to sign and date the consent form included in their baseline pack and return to the research team either in electronic-consent online format or in the pre-paid envelope provided to those who opt for paper-based involvement. Patients who return an incomplete paper consent form will be sent a letter asking them to complete the consent form, along with a copy of their consent form with the incomplete section(s) highlighted and a pre-paid return envelope. Recruitment will be complete when the signed consent form is received at Keele CTU. This recruitment method is similar to a recent successful trial ^59^, and the TREADON pilot and feasibility trial ^34^.

Patients who decline to participate during the telephone call will be thanked for their time and advised that they should contact their GP or referring physiotherapy/podiatry service regarding further care if required.

The clinical researcher taking part in the informed consent process will have received appropriate training and will be authorised in the trial delegation log and permitted to take informed consent. The right of the participant to refuse consent without giving reasons will be respected. Furthermore, the participant will remain free to withdraw from the trial at any time without giving reasons and without prejudicing any further treatment.

***Loss of capacity following informed consent***

Where valid, informed consent is provided by the participant and the participant subsequently becomes unable to give on-going informed consent by virtue of physical or mental incapacity, the initial consent provided remains legally valid and endures. Participants who lose capacity after informed consent has been obtained and are unable to continue with protocol treatment or questionnaires will be withdrawn from further active trial participation.

### Randomisation

Following confirmation of eligibility, receipt of a correctly completed and signed consent form and completed baseline questionnaire, participants will be randomly allocated to an intervention. The randomisation module in the Research Electronic Data Capture (REDCap) database will be configured to handle randomising participants to intervention arms. The required randomisation sequences (random block randomisation with three different block sizes stratified by identification method and geographical region of intervention site) will be uploaded and implemented into REDCap in accordance with Keele CTU Standard Operating Procedures (SOPs). Seven different versions of the sequences will be created in the event of one or more arms being dropped from the trial. This will be managed by selecting a version number within REDCap. A statistician will generate 10 randomisation lists using the Sealed Envelope Web application ( www.sealedenvelope.com). One randomly selected list will then be uploaded to REDCap by a system administrator. Randomisation will be conducted by an authorised administrator (at the intervention site or CTU, depending on service preference) via REDCap. Emergency telephone backup will also be available. Allocation will be concealed from the blinded researchers, as outlined in *Blinding* section below. Participants will be randomised on an equal 1:1:1:1 basis to one of the following interventions:

1. Self-management advice (SMA) booklet (control arm)
2. SMA booklet plus individualised exercises (SMA-exercises)
3. SMA booklet plus pre-fabricated foot orthoses (SMA-orthoses)
4. SMA booklet plus individualised exercises and pre-fabricated foot orthoses (SMA-combined).

The following information will be required for randomisation:

- Participant details, including initials, sex at birth and date of birth
- Participant trial ID
- Name of person undertaking randomisation
- Confirmation of eligibility
- Confirmation of written informed consent and date
- Completed baseline questionnaire including participant recorded pain score
- Identification method and geographical region (stratification variables).

All participants who are randomised will be informed of their allocation via email or in writing by postal mailing. Participants in the SMA control arm will be posted a SMA booklet. Participants in the three clinician-delivered treatment arms will be given a SMA booklet at their first treatment appointment. Where participants have been allocated to a treatment package which includes exercise and/or orthotic device intervention, a coordinator member of the trial team will then liaise with the appropriate service and a first appointment for each trial participant will be made. The service will be responsible for notifying the participant of the date, time and location of their appointment(s). We aim to agree a maximum wait for treatment start of no more than two weeks following randomisation with participating sites. Participants who DNA/UTA appointments will be offered further appointments as needed as part of the trial. The GP of each trial participant will be sent a letter to confirm that their patient is taking part in the research trial. Participants allocated to the SMA booklet control arm will not be required to attend for any trial-specific clinical intervention.

Selection bias at recruitment will be avoided by the use of the following methods: all patients will receive identical trial information and questionnaires minimising the threat to participation bias from the different identification routes, and a clinical researcher will contact patients to discuss eligibility regardless of identification route and will not take part in treatment allocation. Allocation concealment will be achieved by separating the processes and individuals involved in determining treatment allocation and treatment delivery and through the use of random permuted blocks.

### Blinding

It will not be possible to blind participants and treating clinicians (physiotherapists and podiatrists) to treatment allocation. Research staff involved in data collection and analyses (including the trial administrator overseeing collection of SMS text-messages, follow-up questionnaires and minimal data collection; data entry administrator; and lead statistician) will remain blind to treatment allocation until all data collection up to 12-month follow-up has been completed. Statistical analysis of the primary outcome including interim analysis will be carried out by an unblinded trial statistician and separately by the blinded lead statistician with consensus agreement, and independently verified by a blind external statistician collaborator (with expertise in adaptive trials) to ensure the integrity of the trial evaluation.

### Data collection

***Trial assessments***

The schedule of enrolment, interventions and assessments are summarised in extended data. Trial questionnaires have not been included as extended data due to copyrights.

***Follow-up assessments***

**Outcome collection methods and time points**

***Text-messages weekly for 12 weeks, then monthly to month 12***

All participants will be asked to respond to mobile phone SMS text-messages which will be automatically sent each week for 12 weeks, and monthly thereafter to month 12, to collect pain intensity scores. Participants who do not wish to use text-messaging will be offered the option of brief telephone calls at the consent stage.

The research team member will explain this process over the telephone, when gaining informed consent at the start of the participation. Non-responders to the initial text-message will be sent a further automated text-message reminder 24 hours later, those receiving phone calls will receive another call the next working day. Non-response to this text/phone call will be recorded and no further contact at this attempt made.

Over 90% of UK adults have a smartphone ( Mobile phone and internet usage statistics in the UK) and previous research shows that weekly text-messages (followed by a telephone call for the first text-message that is not answered) had an 83% mean response rate ^48, 60^.

***Weekly adherence participant diary for 12 weeks***

All participants randomised to one of the three clinician-supported intervention arms will be asked to keep a weekly paper-based diary over the 12 weeks of the intervention. This will capture aspects of adherence, general participant engagement with the intervention and any self–reported adverse events. The diary can also be used by participants to aid discussions with their treating clinicians. Participants will be asked to return the diary to Keele CTU at 12 weeks, using a pre-paid envelope provided. If after 13 weeks, the participant still has not returned their diary a reminder will be sent by SMS text-message, email or by postal mailing.

***Questionnaire at 12 weeks, 6 months and 12 months***

All participants will be asked to complete a self-report questionnaire 12 weeks, 6 months and 12 months after randomisation. The questionnaire (online or paper) will be sent to participants by Keele CTU at 12 weeks, 6 months and 12 months’ post randomisation. Where a questionnaire has not been returned to Keele CTU after 10 days from link URL in SMS text-message, email or mail despatch, a reminder SMS text-message, email, or postcard will be sent. Where a questionnaire is still not returned following this, a second questionnaire will be sent after 15 days after the initial SMS text-message, email or postcard. For non-responders, minimum data collection (MDC) to capture the primary outcome measures will be undertaken by a Keele trial team member by telephone after 25 days from the initial SMS text-message invite, email or mail despatch. Up to three call attempts will be made within seven days. During this phone call if participants decline to provide MDC over the phone they can be offered the choice to receive an online or postal MDC questionnaire. Where a participant is not able to be contacted by telephone, a MDC questionnaire will be sent through a link URL in an SMS text-message, email or by post. Participants will be asked to return the completed questionnaire (together with the participant diary at 12 weeks) to the Keele CTU, either online or using the pre-paid envelope.

### Withdrawal criteria

Participants may withdraw from the trial at any time without giving reasons and without prejudicing any further treatment. Any information provided up to the point the participant withdraws will be included in the dataset and used unless the participant asks for their data to be destroyed. Discontinuation of exercise or orthoses interventions for any reason is not a reason for withdrawal from the trial.

### Sample size calculation

Up to 696 participants will be required to detect a small-to-moderate standardised between-group effect-size of 0.3 (equating to a minimum clinically important difference of 0.8 in average pain NRS over 6–12 weeks follow-up with anticipated standard deviation 2.7) in at least one clinician-supported intervention arm versus the control arm (SMA booklet alone) with 90% probability, while controlling the probability of the overall type I error at 5% (one-sided) ^61, 62^. The maximum sample size is based on equal allocation of participants, on the assumed, repeated measures correlation of 0.7, baseline-outcome correlation of 0.5 ^63^, 20% loss to follow-up, and 70% response to SMS text-messages (informed by our pilot trial and previous trials). This accounts for multiple comparisons of each of the intervention arms to the control and one formal interim analysis upon the primary outcome being observed for 348 participants (approximately 87 per arm) ^64^. The total sample size of 696 also represents the maximum possible sample size under the two-stage multi-arm multi-stage (MAMS) adaptive trial design; the minimum sample size being half of this if the trial is stopped (all three clinician-supported intervention-control interim evaluations yield between-group differences below the futility bound or at least one beyond the superiority bound) after the interim analysis; hence, the actual sample size will be in the range of 348 to 696. Extensive (>100,000) simulations indicate that the expected sample size varies between 415 and 532 participants. For logistical reasons, recruitment will not be suspended whilst the interim analysis is undertaken; to maintain momentum with recruitment, the trial will carry on recruiting and hence we anticipate, due to the expected lag of around 4 months (12-week data follow-up plus 4 weeks for data transfer, checking and interim analysis), approximately 100 further participants will have been recruited by the time of the interim decision-making. Hence, taking this lag into account, the revised minimum sample size would be about 448 and the expected sample size range would be about 490 to 610. If the efficacy boundary is crossed at the time of the interim analysis and the trial is stopped earlier, the conclusion on the efficacy of this treatment will be based on the test statistic (and the corresponding efficacy boundary value given in *Primary outcome analysis and decisions about adaptions to the trial* section below) at the time of the interim analysis and will not be affected by the data from the participants recruited after the interim analysis. The data from the stage-2 participants, however, in this case, will be included in the primary and secondary analysis to obtain more accurate treatment effect estimates.

### Data analysis plan

A comprehensive data analysis plan (DAP) will be developed and describe all trial analyses. It will be kept as a separate document to this protocol and represents the *a priori* analysis plan. It will be written using SOPs for Keele CTU and an approved version 1.0 will be signed off by the TSC and DMC committees prior to the end of pilot phase recruitment. Consequently, only a brief outline of the analysis plan is provided below.

***Summary of baseline data and flow of participants***

A Consolidated Standards of Reporting Trials (CONSORT) flow diagram, together with appropriate adaptive trial design extensions ^65^, will be produced to document the flow of participants through the trial and will include reasons for withdrawal if given. Serious or trial-related adverse events and protocol violations will be reported throughout the trial by treatment arm. Descriptive statistics will be used to describe the key baseline characteristics of participants included at each stage of recruitment and follow-up. The DMC will receive these data and serious or trial-related adverse events and protocol violations disaggregated by trial arm, prepared by the unblinded trial statistician.

***Primary outcome analysis and decisions about adaptations to the trial***

For the primary estimand (Estimand 1) the main between-group evaluation for the primary outcome will be undertaken using linear mixed modelling adjusted for geographical region treatment site, identification method, age, sex at birth and baseline pain score, by analysing participants according to their randomised allocation (intention-to-treat [ITT]). At stage 1, approximately 87 participants will be recruited to each arm (totalling 348 participants) and the primary outcome obtained: if a clinician-supported intervention arm is shown to be inferior to control it will be dropped for futility i.e. if it is below the futility bound of t=0.000 (if all clinician-supported interventions are inferior [below the futility bound] the trial will be stopped); if one or more clinician-supported interventions are shown to be superior to control the trial will be stopped for efficacy (i.e. standardised mean group comparison test statistic is above 2.352 for at least one of the intervention arms (versus control), then the trial will be stopped and the arm with the highest test statistics will be recommended). If the trial continues, additional participants (approximately 87) will be recruited to each remaining clinician-supported intervention arm and control. At the end of stage 2, the focus of the pain NRS evaluation will be on superiority testing against an efficacy boundary of t=2.218, and ensuring an overall one-sided 5% type 1 error; hence, if the standardised treatment effect statistic is above 2.218, then the corresponding treatment arm will be recommended. The testing boundaries are constructed under a generalised Dunnett testing procedure using a triangular efficacy boundary and binding futility boundary (i.e. if a test statistic for the corresponding treatment is crossing lower futility boundary, the treatment must be dropped at the interim analysis). If the test statistic for at least one of the treatments crosses the upper efficacy boundary at the time of the interim analysis, the trial will be stopped earlier. If the test statistics for more than one treatment cross the upper efficacy boundary (either at the time of interim or final analyses), all corresponding treatments will be concluded to be superior to the control. Interim and final analysis of the primary pain outcome will be carried out by an unblinded trial statistician and separately by the blinded lead statistician, and independently verified by a blind external statistician collaborator (with expertise in adaptive trials) to support scientific integrity.

***Secondary outcome analysis***

Between-group evaluation of secondary outcomes will only be undertaken at the end of the trial. Analysis will be through linear and generalised mixed models with aligned link function appropriate to the outcome data being analysed. Analysis will consider participant-level clustering (repeated measures data) through participant-level random effect and the following fixed effects: geographical region, identification method, age, sex at birth, baseline pain score and corresponding baseline value (as appropriate). Reporting of numbers analysed at each follow-up stage will be given and summary descriptive statistics will be presented per treatment arm that align to the type of data e.g. mean (standard deviation (SD)) and/or median (interquartile range) for numeric outcomes; frequency counts and percent per category per treatment arm for categorical outcome variables.

For all primary and secondary analyses, the main between-group estimates will be given along with 95% two-sided confidence intervals and p-values.

As well as the primary endpoint evaluation of the 6 to 12 week between-group difference in pain intensity, we will also evaluate as secondary analyses the between-group differences at individual time-points between weeks 1 to 12 through inclusion of a time x group interaction term. Follow-up pain NRS trajectories will be presented graphically to show the comparative summary data between treatment arms.

***Subgroup analyses***

Subgroup analyses of the primary pain outcome will also be carried out (including age, sex, ethnic origin, body mass index, time on feet per day as potential modifiers of treatment effect); the full and exact specification of subgroup variables will be decided upon discussion with the TSC. These will be agreed *a priori* with the TSC and a hypothesised direction of effect for these subgroup analyses will be stated in the DAP. In each case, exploratory statistical modelling of the subgroup effect will be through statistical interaction of the dummy treatment variable x subgroup variable.

***Adjusted analysis***

Analyses will consider participant-level clustering (repeated measures data) through participant-level random effect and the following fixed effects: geographical region, identification method, age, sex at birth, baseline pain score and corresponding baseline value (as appropriate).

***Interim analysis and criteria for the premature termination of the trial***

The decision about adaptations to the trial will be made based on Estimand 1. At stage 1, approximately 87 participants will be recruited to each arm: if a clinician-supported intervention arm is shown to be inferior to control it will be dropped for futility i.e. if it is below the futility bound of t=0.000 (if all clinician-supported interventions are inferior [below the futility bound] the trial will be stopped); if one or more clinician-supported interventions are shown to be superior to control the trial will be stopped for efficacy (i.e. standardised mean group comparison test statistic is above 2.352 for at least one of the intervention arms (versus control), and the arm with the highest test statistics will be recommended). Any potential requirement to stop any trial arms for safety reasons will be closely monitored by the DMC.

***Participant population***

All randomised participants will be analysed as randomised. Analysis populations will be defined according to the estimand of interest. For any outcome, participants will be included in an analysis if baseline and at least one follow-up measurement is available for that outcome.

***Procedure(s) to account for missing or spurious data***

The linear and generalised mixed models specified above in the *Secondary outcome analysis* and *subgroup analyses* sections analyse all available data and assume that missingness is ‘missing at random’ rather than ‘missing completely at random’. A sensitivity analysis will also be conducted using a multiple imputation approach where missing data due to an intercurrent event will be differently imputed than missing data due to other reasons. The details of the sensitivity analysis will be included in the DAP.

### Economic evaluation

***Health economic outcomes***

The economic evaluation will determine the cost-effectiveness of a SMA booklet combined with individualised exercises and/or prefabricated foot orthoses versus a SMA booklet alone. Resource use information will comprise participant-specific costs for the type of intervention received including sessions attended and any other resources incurred as part of the intervention. PHP-related resource data will be collected for primary care consultations (e.g. GPs, practice nurses, First Contact Practitioners), visits to other professionals (e.g. hospital consultants, physiotherapists), private healthcare, prescribed medications and hospital-based tests and investigations and procedures, non-drug treatments (e.g. injections) and inpatient stays. All costs will be collected via participant questionnaires at 6 and 12 months. Unit cost data will be derived from nationally represented sources such as the British National Formulary (BNF), the National Schedule for Reference Costs and the Unit Costs of Health and Social Care ^66, 67^. Given the nature of the condition, broader costs related to both out of pocket costs (e.g., over the counter medications) and productivity losses (e.g., time-off work related to PHP and reduced work performance (presenteeism) will be collected. Information on time off work, occupation, typical work activities and the nature of their employment (full time or part time) will be requested, and the Single-Item Presenteeism Question from the Work Productivity and Activity Impairment Questionnaire ^68^ will be used to estimate productivity losses relating to presenteeism. The average wage for each respondent will be identified using UK Standard Occupational Classification coding and annual earnings data for each job type.

***Health economic analysis***

A full health economics analysis plan (HEAP) will be written in parallel with the DAP, prior to the analyses. The plan will be agreed by the trial health economists, and other members of the trial and TSC/DMC prior to the analysis. Consequently, a brief outline of the data analysis plan is included here. An economic analysis will be undertaken alongside the trial to estimate the cost-effectiveness of the interventions in the final analysis and will adhere to the recommendations of the NICE Reference Case and these will be calculated using EQ-5D-5L responses.

The evaluation will take the form of an incremental cost-utility analysis to estimate the cost per quality adjusted life year (QALY) over 12 months follow-up using data from the trial. The base-case analysis will be from an NHS/personal social services perspective, with an additional analysis from a wider societal perspective considering costs of productivity loss. The outcome of interest for the economic analysis is the QALY, and utility data will be estimated using participant responses obtained from the EQ-5D-5L questionnaire administered at all follow-up time points using the “area under the curve” approach. The mapping function developed by the Decision Support Unit, using the ‘Economic Methods of Evaluation of Health and Care Interventions (EEPRU) dataset’, will be used for mapping the data from the EQ-5D-5L to the EQ-5D-3L in the reference-case analysis ^69^. A cost-consequence analysis will initially be reported, describing all the important results relating to costs and consequences (across the full range of clinical and cost outcomes). Subsequently, incremental cost-utility analysis will be undertaken to estimate the incremental cost per QALY gained, adjusting for baseline covariates in line with the primary clinical evaluation. The robustness of the results will be explored using sensitivity analysis.

Final outcomes adjusting for the bias in the health economic analysis results from the adaptive design of the trial will be expressed as incremental cost-effectiveness ratios (ICERs) using ICER plots and cost-effectiveness acceptability curves to represent the probability of being cost-effective at different willingness to pay thresholds. Given the limited consensus in analysing economic evaluations alongside adaptive multi-arm platform trials ^70, 71^, a decision-analytic model will be developed to enable adjustments in the health economic estimates to account for potential imbalances associated with the adaptive design. The model will be populated with data obtained alongside the trial but will be complemented with external data from literature review of previous models, and stakeholder consultations. The model will be subject to deterministic sensitivity and probabilistic sensitivity analyses in the final analysis. Cost-effectiveness planes and cost-effectiveness acceptability curves will be presented to show the probability the intervention is cost-effective at different cost/QALY thresholds. Full details of the model approach will be described in the HEAP.

***Internal pilot phase***

Our pilot and feasibility trial demonstrated feasibility of participant identification and recruitment, intervention fidelity, adherence, and retention at general practices and physiotherapy and podiatry services in the West Midlands in England ^34^. We will undertake an internal pilot phase in the main TREADON trial to test participant identification and recruitment, intervention fidelity, adherence to orthoses and exercises, and response to SMS text-messages across all geographical regions in England and Scotland.

**Objectives:** Specific objectives of the internal pilot phase of the trial are to:

1.   check the numbers of recruited participants overall, per month and per region per month

2.   explore intervention fidelity and participant adherence to exercises and orthoses

3.   determine response to SMS text-messages.

**Methods:** The internal pilot will last for 6 months, commencing from the start of recruitment (month 10 of the trial timeline). All regions will participate in the internal pilot. Data collection methods will be as described above.

**Outcomes:** Outcomes of interest for the internal pilot include:

1.   numbers of adults with plantar heel pain recruited (overall; per month; per region per month)

2.   intervention fidelity measured by percentage of participants who received the allocated intervention

3.   self-reported adherence to exercises and orthoses measured by the percentage reporting performing exercises and/or wearing orthoses as advised

4.   response to SMS text-messages collecting primary outcome pain NRS data during weeks 6–12.

**Sample size:** Sixty participants are required across the three clinician-supported intervention arms to be able to estimate the proportion of participants adhering to exercises/orthoses with at least 80% confidence (lower limit 1-sided alpha 0.2) with a 5% margin of error, assuming adherence of 80%.

**Progression criteria:** A success criteria traffic-light system relating to the internal pilot trial objectives will be used to inform whether we stop at the stage of the internal pilot phase, proceed to the main trial (including the planned interim analyses) or proceed but with protocol amendments ( Table 4).

**Table 4.** Internal pilot progression criteria.

	Do not proceed to main trial (red)	Proceed to main trial with protocol amendments (amber)	Proceed to main trial (green)
Participant recruitment:			
Overall by month 6 of internal pilot recruitment	<78 (<50%)	78–155 (50–99%)	≥156 (≥100%)
Number recruited per month *	<15	15–29	≥30
Number recruited per region per month*	<5	5–9	≥10
Intervention fidelity:			
Received allocated treatment	<80%	80–99%	100%
**Adherence to intervention:**			
Performing exercises/wearing orthoses as advised	<60%	60–79%	≥80%
Follow-up:			
6–12 week SMS text-message response	<50%	50–69%	≥70%

*in months 4-6 of internal pilot allow for phased in recruitment over months 1-4.

### Data handling

***Data collection tools and source document identification***

Self-report questionnaires (online and paper-based), treatment diaries, SMS text-messages and CRFs will form the basis of data collection.

***Data handling and record keeping***

Data management will be carried out in accordance with a Data Management Plan, in accordance with Keele University Health and Social Care Research (HSCR) SOPs. The trial data will be stored on Keele University storage services within the UK and protected by industry standard security tools. All confidentiality arrangements adhere to relevant data protection regulations and guidelines (General Data Protection Regulation (GDPR), Caldicott, General Medical Council (GMC), Medical Research Council (MRC) UK Policy) and the CI and the Data Custodian has responsibility for the use, security and management of all data generated by the study.

Questionnaires completed electronically will be date stamped electronically on receipt at Keele CTU. Paper-based questionnaires will be sent to the Keele CTU data management team in pre-paid envelopes provided to participants. Paper-based questionnaires will be date stamped on receipt at Keele CTU. Questionnaires will then be logged as returned on a management database and the participant’s responses entered into the trial database; the database will be tested *a priori* for reliability. The trial statistician will determine coding of questionnaire items, in accordance with standardised coding procedures of Keele CTU, to facilitate data entry. Members of the research team will enter data and cross checks (a minimum of 1 in 10) will be carried out by other team members to ensure reliability and quality assessment of data entry.

### Ethical and trial administration

This clinical trial has been designed, and will be run, in accordance with the principles of GCP. The lead CI (ER) will ensure there is clear delegation of responsibilities within the trial team and will be supported by the lead academic representative (MJT) and lead representatives from collaborating academic institutions (GHe, TJ, AMK, JK, HBM, NEF). The prefabricated orthoses used in the trial are CE-marked medical devices being used for their intended purpose.

We do not anticipate any major ethical concerns with this trial. All participants will receive at least self-management advice which accords with recommendations in a NICE Clinical Knowledge Summary for PHP ^7^. In addition, some participants will also be randomised to receive individualised exercises and/or prefabricated orthoses which are already available within NHS clinical care. All interventions are deemed safe, with only minor expected adverse events (for example skin irritation from orthoses, muscle soreness from exercise) seen in our pilot and feasibility trial ^34^. Trial safety reporting procedures will ensure any unexpected serious adverse events which are deemed related to the trial will be reported to the Research Ethics Committee, Sponsor, TSC and DMC.

The trial requires the recruitment of patients identified within the NHS to an individually randomised trial involving the collection of primary patient-based data. The CTU will operate this activity in accordance with the Data Protection Act 2018, UK GDPR, other relevant regulations and GCP guidelines. The trial data will be stored on Keele University managed storage services, in a cloud environment hosted by AWS, held within the UK and protected by industry standard security tools. The security of the cloud is the responsibility of AWS, however configuration of the infrastructure and security within the cloud is the responsibility of Keele University. AWS personnel will not have access to the data stored within the cloud unless requested by Keele University or where it is necessary to prevent fraud, abuse or comply with the law. Keele University as the Sponsor has a quality management system in place containing standard operating procedures which will be adhered to in the conduct of the trial.

Potentially eligible patients will receive information about the trial and will have time to consider this prior to undertaking telephone screening and prior to providing written informed consent. Participants will be assured of confidentiality and participant identification details will not be made available to anyone outside the trial team. GPs, physiotherapists and podiatrists will be informed of their patients' willingness to be part of the trial (depending on the initial source of participant identification). Those who do not consent to be part of the trial or are ineligible will be asked for their consent to use the information already provided for the trial and given advice to consult their GP (or physiotherapist/podiatrist if identified from physiotherapy/podiatry referral screen) if their PHP continues to be troublesome.

All data collected during the trial will be handled and stored in line with Keele CTU’s Data Security procedures and SOPs, which are in accordance with the Data Protection Act 2018, UK GDPR, other relevant regulations and GCP guidelines. We will anonymise and archive the data for 10 years on Keele University managed storage services. Lead statistician (ML) will be the data custodian. We will make the data accessible to other researchers, in line with Keele CTU procedures.

***Amendments***

The detailed protocol will be updated in response to approved amendments, as required. All amendments will be reviewed and authorised by the Sponsor and submitted for review or information to the Research Ethics Committee and Health Research Authority, as appropriate.

***Regulatory compliance***

The trial will be conducted in accordance with the principles of GCP in research studies and the UK Policy Framework for HSCR. Keele CTU has a quality management system in place containing standard operating procedures which will be adhered to in the conduct of the trial. Studies run by Keele CTU may be subject to an audit by Keele University as the Sponsor.

### Protocol compliance

Non-compliance may be identified through any trial activity but in particular using central monitoring procedures such as consent form review or data management, CRF review, site visits and self-reporting by the trial site or participants. Deviations from protocols and GCP may occur in research studies. Many of these instances are technical non-compliances that do not result in harm to the trial participants, do not compromise data integrity, or significantly affect the scientific value of the reported results of the trial. These technical deviations will be documented, and appropriate corrective and preventative actions will be taken by the research team with responsibility being taken by the CI and where needed with agreement from the TSC.

### Notification of serious breaches to GCP and/or trial protocol

Participating sites are expected to notify Keele CTU as soon as they become aware of a serious breach. A “serious breach” is a breach which is likely to affect to a significant degree:

- the safety or physical or mental integrity of the participants of the trial; or
- the scientific value of the trial.

These will be reported according to Keele University’s SOPs.

### Data protection and patient confidentiality

All information collected during the trial will be kept strictly confidential. Information will be held securely on paper and managed electronically by Keele University through Keele CTU. Keele CTU complies with data protection regulations:

- appropriate storage, restricted access to disposal arrangements for participants personal and clinical details
- consent from participants for access to their healthcare records by responsible individuals from the research staff or regulatory authorities, where it is relevant to trial participation
- consent from participants for the data collected from the trial to be used to evaluate safety and develop new research
- all data collection forms that are transferred to and from Keele CTU will be coded with a trial number and will include up to three further participant identifiers: the participant’s sex at birth, initials and date of birth
- where anonymisation (and/or appropriate pseudo-anonymised) of documentation is required, participating centres are responsible for ensuring only the instructed identifiers are present before sending to Keele CTU.

All data will be stored on Keele University managed storage services, held within the UK and protected by industry standard security tools. Roles and permissions are applied to users within the network as well as within an application to restrict what data a user can access and operations they can perform. All research staff/CTU operational staff involved in this trial adhere to robust data security procedures and have explicit duties of confidentiality. These practices are written into their employment contracts and are equivalent to the duty placed on NHS staff.

If a participant withdraws consent from further trial intervention and/or further collection of data, their data will remain on file and will be included in the final trial analysis.

### Indemnity

The trial is sponsored by Keele University and covered by Keele University indemnity arrangements.

The NHS has a duty of care to patients treated, whether or not the patient is taking part in a trial, and the NHS organisation remains liable for clinical negligence and other negligent harm to patients under this duty of care.

Agreements between the Sponsor and participating NHS organisations detailing trial conduct and the responsibilities to be honoured by each party will be fully executed before the trial can start at the local NHS Trust/Health Board.

### Post-trial care

Participants allocated to intervention arms including prefabricated orthoses will be allowed to keep their orthoses after the end of the trial intervention period. The assessments and treatments by the physiotherapist or podiatrist will stop once the participant has completed the 12-week intervention period. Participants will not be offered further healthcare as part of their trial involvement once they have been discharged by their treating physiotherapist/podiatrist following their episode of care within the trial. The participants randomised to either exercise or foot orthoses will have the opportunity to continue with the advice and exercises given and/or continue to use the orthoses.

### Access to the final trial dataset

At the end of the trial, archiving of essential trial documents at Keele University will be authorised by the Sponsor following submission of end of trial reports, which will be for 10 years after the end of the trial. Destruction of essential documents will be carried out in accordance with Keele University SOPs.

All data will be held by Keele CTU and will be archived in the designated Keele CTU archive facility. Arrangements for the destruction of all confidential data will be carried out in accordance with Keele University SOPs.

Any subsequent requests for access to the data (and statistical code) from anyone outside of Keele CTU (e.g. collaboration, joint publication, data sharing requests from publishers) will be carried out in accordance with Keele University SOPs.

## Dissemination

Informed by NIHR ‘Push the Pace’ guidance, dissemination of the outputs arising from this research will be supported by the Impact Accelerator Unit within Keele University’s School of Medicine. We will also seek to optimise dissemination to facilitate translation into clinical practice. Results will be made freely available to patients, clinicians and commissioners, by publishing in open-access peer-reviewed journals, on our website, through social media, and presenting at scientific meetings. Dissemination on how to translate the findings into easily understandable messages and on how best to disseminate the results (magazine, newspapers, radio) will be supported by our lay co-applicant (JB) and our patient advisory group. We will share our trial findings with participants via our website, dedicated social media feeds (Facebook, Twitter/X) and posters in participating general practices and physiotherapy and podiatry services. A summary describing the trial findings will be provided to all trial participants.

## Data Availability

No data are associated with this article. Recruitment methods flow charts. Keele Data Repository. Accessible: https://doi.datacite.org/dois/10.21252%2F1724-cw45 ^72^ Participant self-management advice booklet. Keele Data Repository. Accessible: https://doi.datacite.org/dois/10.21252%2Fvwsm-r606 ^73^ Trial-specific exercises. Keele Data Repository. Accessible: https://doi.org/10.21252/6zj5-p633 ^74^ Schedule of enrolment, interventions and assessments table. Keele Data Repository. Accessible: https://doi.org/10.21252/2yfk-bc90 ^75^ Data are available under the term of the Creative Commons Attribution 4.0 International Public License (CC BY-ND 4.0 licence)

## Reporting guidelines

Keele Data Repository ^76^: SPIRIT checklist for ‘Clinical and cost-effectiveness of individualised exercises and foot orthoses in the treatment of plantar heel pain: the TREADON randomised multi-arm multi-stage adaptive trial’. https://doi.org/10.21252/e5r8-hc73

Available under the terms of the CC BY-ND 4.0 licence.
