# Supplementary material for: Clinical and cost-effectiveness of individualised exercises and foot orthoses in the treatment of plantar heel pain: protocol for the TREADON randomised multi-arm multi-stage adaptive trial
Source: NIHR Open Res. 2026 Feb 24;5:42. Originally published 2025 May 6. [Version 2] doi: 10.3310/nihropenres.13930.2 (PMC12141997; doi:10.3310/nihropenres.13930.2)
Supplement: Peer review history

# Reviewer report: Reviewer response for version 1

**DOI:** 10.3310/nihropenres.15138.r35694

**Published:** 2025-06-19

**Authors:** Dylan Morrissey

**Affiliations:** 1 Queen Mary University of London, London, England, UK

Plantar heel pain (PHP) is the most common soft tissue foot condition and impairs mobility, physical function, ability to work, and quality of life. Systematic reviews highlight a need for high-quality randomised controlled trials (RCTs) of exercises and orthoses for PHP.

 Objectives

 To determine the clinical and cost-effectiveness of individualised exercises and/or prefabricated foot orthoses plus self-management advice (SMA) compared to SMA alone in adults with PHP.

 Methods

 This is a multi-centre four-arm two-stage adaptive parallel-group RCT of 696 participants with PHP recurred from primary care. They will be randomised 1:1:1:1 to: (1) self-management advice as a minimal intervention control, (2) SMA plus individualised exercises, (3) SMA plus prefabricated foot orthoses, or (4) SMA plus individualised exercises and prefabricated foot orthoses.

 The outcomes are pain at 6-12 weeks and cost-effectiveness at 12 months.

 There are innovative approaches to recruitment, analysis, adaption and measurement.

 PPIEP has assisted meaningful design decisions.

 This is a well-designed trial and will generate useful data in an under-researched population.

 I have no major concerns with the design or trial team.

 Some minor comments:

 The rationale for researching PHP is well made in the introduction.

 The figure of 10% incidence could do with more detail.

 It would be useful to consider footwear in aetiology.

 A mention of differential diagnoses in the background would also be helpful.

 The exercise section could include aerobic fitness.

 The section in the introduction about the effects of minimal interventions is well worth expanding somewhat. This will be critical information to come from the trial as well as the intervention effects.

 The decision not to treat with shockwave therapy requires more thought as it has arguably the strongest current evidence.

 I find the end point of 6 to 12 weeks to be imprecise and surprising. Given that this is a problem which lasts for many years for many patients, surely a longer-term outcome would be more beneficial. Why was this chosen?

 Also good to justify the choice of primary outcome measure. What is the justification for an average pain numeric rating scale, especially given that first step pain is  pathognomic.

 How will the impact of weekly telephone calls be considered, as this intensity of contact may change participants interaction with the interventions.

 I do wonder how EDI will be considered and equity assured. This is a common condition in populations where the cultural norm is flat or no footwear indoors. The SMA booklet could readily be made available in more languages, and possibly culturally adapted.

 Therapist Fidelity throughout the trial might need tone addressed given likely turnover.

 The recruitment process is intensive and innovative. I do wonder if recruiting through local pharmacies could be useful.

Is the study design appropriate for the research question?

Yes

Is the rationale for, and objectives of, the study clearly described?

Yes

Are sufficient details of the methods provided to allow replication by others?

Yes

Are the datasets clearly presented in a useable and accessible format?

Partly

Reviewer Expertise:

Sports, exercise and musculoskeletal physiotherapy.

I confirm that I have read this submission and believe that I have an appropriate level of expertise to confirm that it is of an acceptable scientific standard, however I have significant reservations, as outlined above.

# Reviewer report: Reviewer response for version 1

**DOI:** 10.3310/nihropenres.15138.r35542

**Published:** 2025-06-06

**Authors:** Finn Johannsen

**Affiliations:** 1 Bispebjerg Hospital, Copenhagen, Denmark

Interesting practical clinical study.

 I look forward to see the results of this study.

 My only concern is the very broad inclusion criteria, and only arthritis being an exclusion. What about diabetes, hypercholesterolemia, psoriasis, tarsal tunnel syndrome, n.suralis syndrome, calcaneus stress fracture, heel fat pad syndrome.

 I would recommend stratification, for BMI above 30, sex, and maybe pain.

Is the study design appropriate for the research question?

Yes

Is the rationale for, and objectives of, the study clearly described?

Yes

Are sufficient details of the methods provided to allow replication by others?

Yes

Are the datasets clearly presented in a useable and accessible format?

Yes

Reviewer Expertise:

Rheumatology.

I confirm that I have read this submission and believe that I have an appropriate level of expertise to confirm that it is of an acceptable scientific standard.

# Reviewer report: Reviewer response for version 1

**DOI:** 10.3310/nihropenres.15138.r35689

**Published:** 2025-05-28

**Authors:** Mohammad Daher

**Affiliations:** 1 Brown University, Providence, Rhode Island, USA

This article is an extensive and well developed protocol for the TREADON randomized controlled trial looking at outcomes of patients with plantar fasciitis (or PHP) undergoing different management techniques (1-SMA,  SMA and orthoses, SMA and individualized exercises, SMA and orthoses and exercises). They plan to look at functional outcomes as well as cost effectiveness. Cost-effectiveness is extremely important as it is lacking in the literature.

 This protocol goes through all the necessary requirements for the RCT to be conducted in a successful and ethical and regulation-compliance manner.

 I only have 2 comments:

 -it would be beneficial if you can provide the readers with a copy of the SMA booklet as an appendix

  -Are you planning to do a ITT analysis or per protocol analysis, or both. I recommend both.

Is the study design appropriate for the research question?

Yes

Is the rationale for, and objectives of, the study clearly described?

Yes

Are sufficient details of the methods provided to allow replication by others?

Yes

Are the datasets clearly presented in a useable and accessible format?

Yes

Reviewer Expertise:

Orthopedics

I confirm that I have read this submission and believe that I have an appropriate level of expertise to confirm that it is of an acceptable scientific standard, however I have significant reservations, as outlined above.

# Reviewer report: Reviewer response for version 1

**DOI:** 10.3310/nihropenres.15138.r35690

**Published:** 2025-05-28

**Authors:** Keith Rome

**Affiliations:** 1 Auckland University of Technology, Auckland, New Zealand

Thank you for the opportunity to review a novel and innovative clinical trial on the clinical and cost effectiveness of individualised exercises and prefabricated foot orthoses for people with plantar heel pain (PHP). The proposed clinical trial is a multi-centre four-arm two-stage adaptive parallel-group clinical trial with internal pilot of 696 participants with PHP.  Participants will be identified from general practice, physiotherapy/podiatry referrals and self-referral, and randomised to four groups: (1) self-management advice (SMA - control), (2) SMA plus individualised exercises, (3) SMA plus prefabricated foot orthoses, or (4) SMA plus individualised exercises and prefabricated foot orthoses. Outcomes will be collected by SMS text-message (weekly during weeks 1–12, monthly during months 4–12) and questionnaires at 12 weeks and 6 and 12 months. The primary outcome is change in PHP intensity (0–10 numeric rating scale) between baseline and the average over 6–12 weeks. There are a number of secondary outcomes reported.

 Overall, the proposal is very well written. There is a dearth of evidence on the cost effectiveness of any podiatric interventions. The authors have provided a very sound approach that has the potential to have a significant impact on current practice and policy.

 I have no major concerns with the current proposal. However, I have a number of comments that the team may want to reflect on before proceeding.

1. There are numerous secondary outcomes that participants need to complete.  Participants may be overwhelmed with the number of questionnaires and may not complete them or just report a neutral response. Currently, the following outcome measures are being reported: PHP intensity score, first step pain, Foot Function Index, (pain, disability, activity restriction subscales, and overall), patient global rating of change, pain self-efficacy questionnaire36, brief illness perceptions questionnaire, quality of life (EuroQol 5-Dimension 5-level instrument (EQ5D-5L), work loss and presenteeism, self-reported healthcare use for PHP (NHS and private), self-reported treatment adherence, treatment credibility and satisfaction with care.
2. Footwear. Both from a clinical and cost-effectiveness perspective, will participants be required to purchase new footwear to accommodate the new foot orthoses?
3. Foot orthoses require appropriate footwear to ensure they fit.  Seasonal variation may impact the use of enclosed footwear. For example, on hot, humid days participants may not be able to use foot orthoses in open-type footwear. Perhaps the authors should advice participants of this issue.
4. Once the trial has been completed will all participants receive the full package that includes SMA plus individualised exercises and prefabricated foot orthoses? 

Is the study design appropriate for the research question?

Yes

Is the rationale for, and objectives of, the study clearly described?

Yes

Are sufficient details of the methods provided to allow replication by others?

Yes

Are the datasets clearly presented in a useable and accessible format?

Yes

Reviewer Expertise:

Professor of Podiatry with nearly 40 years of podiatric research experience within rheumatology and long term chronic foot conditions.

I confirm that I have read this submission and believe that I have an appropriate level of expertise to confirm that it is of an acceptable scientific standard.
