# Supplementary material for: Clinical and cost-effectiveness of individualised exercises and foot orthoses in the treatment of plantar heel pain: protocol for the TREADON randomised multi-arm multi-stage adaptive trial
Source: NIHR Open Res. 2025 May 6;5:42. [Version 1] doi: 10.3310/nihropenres.13930.1 (PMC12141997; doi:10.3310/nihropenres.13930.1)
Supplement: Peer review history

# Reviewer report: Reviewer response for version 1

**DOI:** 10.3310/nihropenres.15138.r35536

**Published:** 2025-06-05

**Authors:** Lindsey Cherry, Dr Jill Halstead-Rastrick

**Affiliations:** 1 University of Southampton, Southampton, England, UK; 2 Leeds Community Healthcare NHS Trust, Leeds, England, UK

Overall, this is a well designed trial, likely to lead to clear indication of outcome, of clinical value, which is clearly described in the protocol.

 We note that the protocol for pilot and feasibility has been published previously (refer 1). Clear summary of differences between the previous protocol and full trial protocol could be helpful, in particular differences/adaptations made as a result of prior work; it appears as though the full trial is underway.

 Clarity of/ differentiation between pre-trial single-site pilot versus internal feasibility pilot proposed within the full trial would be of value.

 Further justification for the proposed primary end point is needed; proposed is use of an average pain score generated between 6-12 weeks. This is a fairly long period of time and there is not a clear justification in the protocol (why was this chosen over say 10-12 weeks?). We recognise that average scores are proposed to account for fluctuation in pain however, the current approach is likely to underestimate potential magnitude of change. The fluctuation likely within the 6-week outcome period could result in significant ‘regression to the mean’, losing potentially meaningful sensitivity in data and the identification of pain spikes which could be clinically meaningful. Elaboration of how estimates of meaningful clinical difference will be determined is welcomed.

 PPIE is appropriate and examples of response to feedback are included.

 Ethics are appropriate.

 Overall, this is a well designed trial, likely to lead to clear indication of outcome, of clinical value, which is clearly described in the protocol.

 We note that the protocol for pilot and feasibility has been published previously ( refer 1). Clear summary of differences between the previous protocol and full trial protocol could be helpful, in particular differences/adaptations made as a result of prior work; it appears as though the full trial is underway.

 Clarity of/ differentiation between pre-trial single-site pilot versus internal feasibility pilot proposed within the full trial would be of value.

 Further justification for the proposed primary end point is needed; proposed is use of an average pain score generated between 6-12 weeks. This is a fairly long period of time and there is not a clear justification in the protocol (why was this chosen over say 10-12 weeks?). We recognise that average scores are proposed to account for fluctuation in pain however, the current approach is likely to underestimate potential magnitude of change. The fluctuation likely within the 6-week outcome period could result in significant ‘regression to the mean’, losing potentially meaningful sensitivity in data and the identification of pain spikes which could be clinically meaningful. Elaboration of how estimates of meaningful clinical difference will be determined is welcomed.

 PPIE is appropriate and examples of response to feedback are included.

 Ethics are appropriate.

 Overall, the trial design is appropriate and likely to lead to a clear trial outcome.

 A clear justification for weekly text messaging would be of value. This justification could expand to further explain choice of average pain score rather than single pain score at completion of intervention.

 A clear description of control and comparator arms is given. Figure one is helpful.

 The summary flow chart is sufficiently detailed, supported by text, to guide in a reproducible way the issue of orthoses.

 A rationale for orthotic design is proposed. However, it is not clear why some characteristics (e.g., heel raise) were omitted, especially if considered part of typical standard care. It would be helpful to also include rationale for exclusion of key characteristics alongside inclusion.

 Trial training appears thorough and appropriate.

 Blinding and randomisation are appropriate and well described.

 We note the teams’ acknowledgement that 90% of UK adults have access to a smartphone. However, there is evidence that foot pain is prevalent in people from areas of higher deprivation, with lower educational attainment, and amongst those who are likely to be digitally excluded. It may be helpful to consider Index of Multiple Deprivation adjustment when describing the participant characteristics and when completing analysis. It would also be of value to consider what further measures could be taken to enable research inclusivity within the trial. 

 No consideration is given to duration of heel pain as an inclusion/exclusion criteria. However, acute (e.g., less than 4 weeks) versus chronic pain may have different biopsychosocial drivers that could significantly alter intervention response. This is not explicitly stated as a criterion for sub-group analysis, however we recognise that it may be included / informed by TSC.

 The intervention appears heavily weighted toward addressing biological (mechanical) factors with some inclusion of patient education. However, greater consideration to psychosocial and behavioural factors in future work may be of value.

Is the study design appropriate for the research question?

Yes

Is the rationale for, and objectives of, the study clearly described?

Yes

Are sufficient details of the methods provided to allow replication by others?

Yes

Are the datasets clearly presented in a useable and accessible format?

Not applicable

Reviewer Expertise:

Our combined areas of expertise include research design, delivery and conduct; Podiatry including specialist expertise in MSK/ RMD foot health; service delivery; personalised care; education.

We confirm that we have read this submission and believe that we have an appropriate level of expertise to confirm that it is of an acceptable scientific standard.

## References

[1] Self-management advice, exercise and foot orthoses for plantar heel pain: the TREADON pilot and feasibility randomised trial - PubMed.10.1186/s40814-021-00808-0PMC801503333795024
